# Supplementation of *Bacillus cereus* Enhances Anti-Colitis Effects of Triple Probiotics in Mice: Correlative Evidence for a Gut Microbiota–Spermidine-Related Axis in Quadruple-Probiotic Protection

**DOI:** 10.3390/nu18193164

**Published:** 2026-09-25

**Authors:** Jixiang Li, Xuxu Du, Jing Zhao, Wutang Li, Qian Wang, Lu Zhang, Ming Li, Yuyuan Li

**Affiliations:** 1 Advanced Institute for Medical Sciences, Dalian Medical University, Dalian 116044, China; 15903443766@163.com (J.L.); duxuxu0000002025@163.com (X.D.);; 2 College of Basic Medical Science, Dalian Medical University, Dalian 116044, China; zhaojing010511@163.com; 3 CapitalBio Technology (Liaoning) Co., Ltd., Dalian 116016, China; 4 Hangzhou Grand Biologic Pharmaceutical Inc., Hangzhou 310019, China

**Keywords:** ulcerative colitis, probiotics, *Bacillus cereus*, gut microbiota, spermidine

## Abstract

**Background:** Ulcerative colitis (UC) is a chronic relapsing inflammatory bowel disease associated with gut microbiota dysbiosis. The triple-probiotic mixture BLE (*Bifidobacterium infantis*, *Lactobacillus acidophilus*, *Enterococcus faecalis*) contains highly oxygen-sensitive strains prone to viability loss during gastrointestinal transit. Adding facultative-aerobic *Bacillus cereus* generates a four-strain probiotic formulation BLEB. This study aimed to determine whether adding *B. cereus* improves the anti-colitis capacity of BLE and explore the underlying microbiota–spermidine-related regulatory mechanism. **Methods:** Acute dextran sulfate sodium (DSS)-induced colitis mouse models were applied to compare the therapeutic effects of BLE and BLEB. We evaluated disease phenotype, intestinal epithelial barrier integrity, and splenic Treg/Th17 immune homeostasis. Dynamic 16S rRNA sequencing and untargeted intestinal metabolomics characterized gut microbial and metabolic alterations, with Spearman correlation for microbiota–metabolite association analysis. Transcriptomic profiling was performed on DSS-injured Caco-2 cells to examine molecular responses to spermidine. **Results:** BLEB exerted superior dose-dependent anti-colitis effects compared with BLE. BLEB enriched beneficial *Lactobacillaceae* and restored luminal polyamine metabolites including spermidine. Spearman correlation revealed significant positive correlations between *Lactobacillaceae*-affiliated genera and polyamine levels. In vitro, spermidine reversed DSS-provoked transcriptional perturbations and upregulated the transcription of glutathione-centered antioxidant-related genes in Caco-2 cells. **Conclusions:** Ecological synergy introduced by *B. cereus* may reshape gut microbiota and increase microbiota-related spermidine, partially contributing to the enhanced mucosal protection conferred by BLEB. This work provides pre-clinical evidence supporting the development of aerobic–anaerobic combined probiotic cocktails for auxiliary UC therapy.

## 1. Introduction

Ulcerative colitis (UC) is a chronic, recurrent, non-specific inflammatory bowel disease (IBD). Its core pathological manifestations include intestinal epithelial damage, mucosal immune dysfunction, and severe intestinal microbiota dysbiosis [1,2]. This condition severely impairs patients’ quality of daily life and significantly increases the long-term risk of developing colorectal lesions [3]. Traditional clinical interventions such as aminosalicylates, glucocorticoids, and immunosuppressants often trigger notable systemic adverse effects, including gastrointestinal intolerance, endocrine disorders, and bone marrow suppression [4,5]. Additionally, these conventional treatment regimens fail to fundamentally restore a balanced intestinal microbiome to reverse disease progression [6]. Therefore, there remains an urgent clinical need for safe adjunctive therapeutic strategies targeting intestinal microbiota homeostasis.

Numerous studies have confirmed that gut microbiota imbalance is the core pathogenic driving factor of UC development [7,8]. Once the intestinal homeostasis collapses, the integrity of the tight junctions of the epithelium is compromised, leading to increased intestinal permeability and allowing Gram-negative bacterial lipopolysaccharides (LPSs) to enter the systemic circulation. The circulating LPS subsequently activates the innate immune signaling pathway and triggers the massive secretion of pro-inflammatory mediators including IL-1β, IL-6, and TNF-α [9,10]. In parallel, human/model studies with colitis show a marked shift in the ratio between anti-inflammatory regulatory T cells (Tregs) and pro-inflammatory Th17 cells within the intestinal lamina propria, which further exacerbates mucosal inflammatory infiltration and tissue injury [11,12]. Collectively, microbiota disturbance, epithelial barrier dysfunction, and immune dysregulation form a self-amplifying vicious loop, making microbiota reconstruction a promising therapeutic direction for UC intervention [13,14].

Triple-probiotic mixtures consisting of *Bifidobacterium infantis*, *Lactobacillus acidophilus*, and *Enterococcus faecalis* (BLE) are widely used for gastrointestinal disorders, yet all three strains are highly oxygen-sensitive and susceptible to oxygen-provoked viability impairment during transit through the oxygen-rich upper digestive tract [15,16]. Adding facultative aerobic *Bacillus cereus* to this triple combination constructs a four-strain probiotic formulation (BLEB) [17,18]. Existing microbiological evidence has confirmed that *B. cereus* can consume intraluminal free oxygen and build an anaerobic microhabitat conducive to the colonization of oxygen-sensitive bifidobacteria and lactobacilli [19]. A previous study systematically compared the in vivo efficacy of BLE and BLEB in chronic DSS-induced colitis, and proved that the quadruple formula exerted stronger protective effects on intestinal multi-barriers [20]. Nevertheless, this prior work merely evaluated basic phenotypic parameters and gut microbiota at a single time point, lacking exploration of dose-dependent effects, Treg/Th17 immune homeostasis, and microbial functional metabolites that underpin the synergistic advantages of four-strain probiotics.

Spermidine is a core polyamine metabolite produced by symbiotic lactobacilli, which has received increasing attention due to its comprehensive intestinal protective function [21,22]. Existing data indicate that spermidine can enhance the tight junction structure of the epithelium, alleviate intestinal oxidative stress, and stabilize the Treg/Th17 immune balance [23,24]. It has also been demonstrated to mitigate DSS-induced colitis in mice by modulating intestinal inflammation and gut microbiota homeostasis [25]. Despite these documented benefits, it has not been experimentally validated whether adding *B. cereus* enabled quadruple BLEB to reshape the gut microbiota and increase endogenous levels of intestinal spermidine to achieve enhanced anti-colitis effects.

In the present study, we adopted a two-stage experimental design to address the above limitations. We first compared the anti-colitis efficacy of triple BLE and quadruple BLEB probiotics in an acute DSS colitis model to identify the more effective formulation. We hypothesized that the addition of *B. cereus* enables BLEB to exert enhanced intestinal protection by reshaping gut microbiota and elevating endogenous spermidine levels. Subsequent multi-omics and cellular assays were carried out to verify this microbiota–spermidine regulatory axis, aiming to provide experimental support for the clinical application of compound probiotics containing aerobic strains as auxiliary therapy for UC.

## 2. Materials and Methods

### 2.1. Reagents and Materials

Triple-probiotic mixture (BLE) containing *B. infantis* (GMCC 0460.1), *L. acidophilus* (GMCC 0460.2), and *E. faecalis* (GMCC 0460.3), and quadruple probiotic mixture (BLEB) supplemented with facultative aerobic *B. cereus* (GMCC 0460.4) were provided by Hangzhou Yuanda Biopharmaceutical Co., Ltd. (Hangzhou, China). Based on viable plate counting of the original tablet, the strain ratio of viable bacteria was *B. infantis*:*L. acidophilus*:*E. faecalis*:*B. cereus* ≈ 75:67:11:1. The BLE suspension contained the three anaerobic-origin strains reconstituted from the original tablet formulation by removing *B. cereus* component. BLEB represented the complete four-strain reconstituted tablet suspension. Dextran sulfate sodium (DSS) was obtained from MP Biomedicals. Primary antibodies against Occludin (27260-1-AP), Claudin-1 (13050-1), and GAPDH (10494-1-AP) were obtained from Proteintech; anti-MUC-2 (R014045) from Yamei Biopharmaceutical Technology Co., Ltd. (Shanghai, China); and anti-E-cadherin (EM0502) from HUABIO (Hangzhou, China). HRP-conjugated secondary antibodies (AS014, AS003) were supplied by ABclonal (Wuhan, China). Flow cytometry fluorescent antibodies including FITC-CD4, APC-IL-17A, PE-CD3, and APC-Foxp3 were purchased from BioLegend (San Diego, CA, USA). TRIzol, reverse transcription and qPCR kits were commercially available. Human Caco-2 colon epithelial cell line was preserved in our laboratory. Spermidine standard was purchased from Sigma-Aldrich (St. Louis, MO, USA).

### 2.2. Animals and Ethical Approval

Male ICR mice aged 6 weeks with an average body weight of approximately 22 g were purchased from Liaoning Changsheng Biotechnology Co., Ltd. (Benxi, China). Mice were housed in the Laboratory Animal Center of Dalian Medical University under standardized conditions: 12 h light/dark cycle, ambient temperature 20–26 °C, and relative humidity 40–70%, with free access to standard pelleted chow and sterile drinking water. All animal experimental protocols were reviewed and approved by the Animal Ethics Committee of Dalian Medical University (Ethics No.: XL2604110231). After 7 days of adaptive feeding, mice were randomly assigned to five experimental groups with 7 mice initially per group for phenotypic screening. One animal from the DSS group died during DSS challenge. For colon-length measurement and 16S rRNA microbiota sequencing, *n* = 7 biological replicates were available for each group; fecal samples for microbiota profiling were collected before the death of this animal. The deceased animal was excluded from body-weight monitoring, DAI scoring, serum ELISA, qRT-PCR and untargeted metabolomics, resulting in *n* = 6 per group for these assays. A subset of 3 randomly selected biological replicates from each group was further used for histopathological evaluation, flow cytometry and Western blot analysis.

### 2.3. Model Establishment and Drug Intervention

After one week of acclimatization, the mice were randomly divided into five groups (*n* = 7 per group). The control (Con) group received regular drinking water and daily sterile saline gavage. The DSS model group was supplied with 2.5% (*w*/*v*) DSS drinking water and an equal volume of saline by gavage. The triple-probiotic (BLE) group was given 2.5% DSS water plus daily gavage of BLE suspension at the human equivalent clinical dose. Two quadruple-probiotic groups were included: low-dose BLEB (BLEB-L) and high-dose BLEB (BLEB-H). BLE and BLEB suspensions were freshly prepared with sterile saline before each administration. Dosing was calculated using the FDA-recommended body surface area conversion factor of 12.3, based on the standard adult clinical dose of 0.075 g/kg/day, yielding a mouse equivalent dose of 0.92 g/kg (23 mg daily for a 25 g mouse) for BLE (4.02 × 10^7^ CFU) and BLEB-L (1.7 × 10^7^ CFU). The BLEB-H group received three times this standard equivalent dose (2.77 g/kg, 69.2 mg per 25 g mouse, 5.11 × 10^7^ CFU). All animals received a fixed 0.25 mL gavage volume once daily for 7 consecutive days alongside DSS exposure. Full phenotypic assessments were performed on all five groups to compare the efficacy of BLE and BLEB, while 16S rRNA sequencing, untargeted metabolomics, and Caco-2 transcriptomic analyses were only conducted on Con, DSS, BLEB-L, and BLEB-H samples to focus mechanistic exploration on the more potent quadruple formulation under limited experimental resources.

### 2.4. Body Weight and Disease Activity Index (DAI)

Mouse body weight was recorded every 24 h during the whole modeling period, and the daily Disease Activity Index (DAI) was calculated comprehensively according to three indicators including weight loss, stool consistency and rectal bleeding. Specifically, weight loss was scored as 0 for less than 1%, 1 for 1–5%, 2 for 5–10%, 3 for 10–20% and 4 for over 20%; stool status was rated 0 for normal pellets, 2 for loose stools and 4 for watery diarrhea; rectal bleeding was graded 0 for no blood, 2 for occult blood and 4 for gross bloody stool, and the final total DAI value was the sum of the three individual item scores.

### 2.5. Colon Length and Histopathological Evaluation

On day 8, all mice were humanely sacrificed. The whole colon was separated, rinsed with pre-cooled PBS, and the colon length was measured. Colon tissues were fixed in 4% paraformaldehyde overnight, followed by gradient ethanol dehydration, xylene clearing, paraffin embedding and continuous sectioning (4–6 μm). Hematoxylin–eosin (H&E) staining was performed to observe mucosal crypt structure and inflammatory infiltration, and blinded histopathological scoring was conducted.

### 2.6. Detection of Serum Inflammatory Factors and Lipopolysaccharide (LPS)

Blood was collected from abdominal aorta and centrifuged to isolate serum. The levels of pro-inflammatory cytokines IL-1β, IL-6, TNF-α, and endotoxin LPS in serum were quantified using commercial ELISA kits from Elabscience Biotechnology Co., Ltd., Wuhan, China strictly following manufacturer’s instructions.

### 2.7. Western Blot

Colon tissue total protein was extracted using RIPA lysis buffer containing protease inhibitor cocktail. Protein concentration was quantified by BCA assay. Equal amounts of protein were separated by SDS-PAGE electrophoresis and transferred to PVDF membranes. After blocking with 5% skim milk, membranes were incubated with primary antibodies overnight at 4 °C, followed by HRP-secondary antibodies at room temperature for 1 h. Target bands were visualized with ECL chemiluminescence reagent, and relative protein expression was quantified by ImageJ software ImageJ 1.54k (National Institutes of Health, Bethesda, MD, USA) with GAPDH as loading control.

### 2.8. Quantitative Real-Time PCR (qRT-PCR)

Total RNA was extracted from colon tissues with TRIzol reagent, RNA concentration and purity were determined, and reverse transcription was conducted to synthesize complementary DNA (cDNA). The relative mRNA expression of tight junction and mucin genes (E-cadherin, Occludin, MUC-2, Claudin-1) was detected via qPCR using the 2^−ΔΔCt^ method, with GAPDH as internal reference gene. The detailed forward and reverse primer sequences are as follows: MUC-2 (Forward: 5′-TTCTACGGCCCAGGTGAACT-3′, Reverse: 5′-TTGGTTGGTGTGCTGAGTGT-3′), E-cadherin (Forward: 5′-GTCCTGGGCAGAGTGAGATT-3′, Reverse: 5′-TGGAGCTTTAGATGCCGCTT-3′), Occludin (Forward: 5′-CCCTCTTTCCTTAGGCGACA-3′, Reverse: 5′-CCCAAGATAAGCGAACCTGC-3′), Claudin-1 (Forward: 5′-CCCCATCAATGCCAGGTATGAA-3′, Reverse: 5′-AGGTTGTTTTCCGGGGACAG-3′), GAPDH (Forward: 5′-AGGTCGGTGTGAACGGATTTG-3′, Reverse: 5′-TGTAGACCATGTAGTTGAGGTCA-3′).

### 2.9. Flow Cytometry Analysis of Splenic Treg/Th17 Balance

Spleens were isolated and cut into fragments, digested and filtered through 100 μm cell strainers to prepare single-cell suspension. Red blood cell lysis buffer was added to remove erythrocytes. For intracellular and nuclear staining, cells were stimulated with PMA, ionomycin and brefeldin A, then stained with surface marker CD4, followed by fixation and permeabilization. Intracellular IL-17A (Th17 marker) and nuclear Foxp3 (Treg marker) were labeled by corresponding fluorescent antibodies. Flow cytometry detection was performed, and the proportions of CD4^+^Foxp3^+^ Treg cells and CD4^+^IL-17A^+^ Th17 cells were analyzed by NovoExpress software (v2.2.0), and then Treg/Th17 ratio was calculated.

### 2.10. 16S rRNA High-Throughput Sequencing of Gut Microbiota

Fresh fecal samples were collected from each group on day 4 and day 8 of intervention, snap-frozen in liquid nitrogen and stored at −80 °C. Fecal genomic DNA was extracted using E.Z.N.A.^®^ Fecal DNA Kit (Omega Bio-tek, Inc., Norcross, GA, USA). The V3-V4 hypervariable region of bacterial 16S rRNA gene was amplified by barcode primers 341F/806R. Purified amplification products were used to construct sequencing libraries, and sequencing was performed on Illumina NovaSeq 6000 platform (Illumina, Inc., San Diego, CA, USA). Bioinformatic analysis included α-diversity indices (Chao-1), principal component analysis (PCA) for β-diversity, and differential abundance analysis of microbial taxa at genus and species levels.

### 2.11. Untargeted LC-MS Metabolomics of Intestinal Contents

Colonic contents were thawed gradually on ice. Each 50 mg sample was extracted with 53% methanol solution and subjected to ultrasonic crushing and centrifugation. Pooled quality-control (QC) samples were generated by mixing equal aliquots of all sample supernatants and injected at regular intervals to monitor instrument stability; solvent blanks without intestinal material were inserted between sample batches to assess carry-over contamination. Supernatants were injected into Vanquish Flex UPLC coupled with Q Exactive HF-X mass spectrometer for untargeted metabolomic detection. Chromatographic separation utilized an Acquity UPLC HSS T3 column (Waters Corporation, Milford, MA, USA) (100 mm × 2.1 mm, 1.8 μm) maintained at 40 °C, with mobile phase A (0.1% formic acid aqueous solution) and mobile phase B (0.1% formic acid acetonitrile-methanol mixed solution), at a flow rate of 400 μL/min, and injection volume of 2 μL, as well as the following gradient elution conditions: 0–0.5 min, 5% B; 0.5–5.5 min, 5–50% B; 5.5–9.0 min, 50–95% B; 9.0–10.5 min, 95% B; 10.5–12.0 min, 95–5% B. MS data were acquired under positive and negative ESI ion modes with *m*/*z* scan range 50–1200. Raw LC-MS data were processed for peak picking, retention-time alignment and intensity normalization. Metabolite identification was annotated against HMDB, GNPS, and MoNA databases. Metabolite intensities are semi-quantitative relative values from untargeted metabolomics. Spearman correlation analysis was performed to explore the correlation between differential gut microbiota and spermidine and other core metabolites. KEGG pathway enrichment was carried out for differential metabolites.

### 2.12. RNA-Seq Transcriptomic Verification in DSS-Induced Caco-2 Injury Model

Caco-2 cells were cultured in DMEM medium supplemented with 20% fetal bovine serum at 37 °C, 5% CO_2_ incubator. Three experimental cell groups were set: control group (normal culture), DSS injury group (3% DSS treatment for 24 h), and spermidine intervention group (200 μM spermidine co-treated with 3% DSS). A spermidine-only group without DSS challenge was not included, as the transcriptomic assay aimed to investigate spermidine-mediated transcriptional rescue under DSS-induced injury, rather than spermidine effects on uninjured Caco-2 cells. Total RNA of cells was extracted, and strand-specific sequencing libraries were constructed and sequenced on Illumina NovaSeq platform. DESeq2 software (v1.50.2) was used to screen differentially expressed genes (DEGs, |log_2_FC| ≥ 1, adjusted *p* < 0.01). KEGG pathway enrichment analysis was conducted to explore the protective molecular pathways of spermidine against intestinal epithelial injury.

### 2.13. Statistical Analysis

All data were expressed as mean ± standard error of the mean (SEM). GraphPad Prism 10 software was used for statistical analysis. For longitudinal repeated measurements of body weight and disease activity index (DAI), two-way repeated-measures ANOVA followed by Tukey’s post hoc multiple comparison test was used to evaluate differences across groups and time points. For other endpoint indicators (including colon length, histopathological score, inflammatory cytokine levels, and relative abundance of microbiota), one-way analysis of variance (ANOVA) followed by Tukey’s multiple comparison test was adopted for intergroup comparison. In addition, Spearman correlation analysis was performed to explore the correlation between differential gut microbiota and metabolite profiles. *p* < 0.05 was defined as statistically significant.

## 3. Results

### 3.1. Phenotypic Screening Identifies Superior Anti-Colitis Efficacy of BLEB over Triple BLE Probiotics

We first conducted comprehensive phenotypic evaluation to compare the anti-colitis effects of triple BLE and quadruple BLEB formulations in acute DSS colitis mice (Figure 1A). During the 7-day intervention period, the DSS challenge caused persistent body weight loss and drastically elevated DAI scores. The triple BLE group achieved mild relief of colitis symptoms, while both low-dose (BLEB-L) and high-dose (BLEB-H) quadruple probiotics produced much more obvious improvements, with the high-dose group showing the optimal therapeutic effect (Figure 1B,C). Macroscopic observations of colon tissues and perianal manifestations revealed prominent pathological abnormalities in the DSS group, including obvious anal erythema, swelling, and visible bloody stool. The colon tissues isolated from DSS-treated mice were markedly shortened and atrophic with thin, fragile intestinal walls and severe mucosal congestion. The BLE group displayed mild relief of these gross lesions, while both BLEB-L and BLEB-H groups presented remarkably alleviated perianal hemorrhage and colon shrinkage, with high-dose BLEB showing the most intact intestinal macroscopic morphology (Figure 1D,E). H&E staining revealed severe crypt loss, mucosal erosion, and extensive inflammatory infiltration in the DSS group. The BLE group exhibited partial alleviation of pathological damage, yet BLEB treatment significantly reduced inflammatory infiltration and preserved mucosal structure, accompanied by much lower histopathological scores (Figure 1F). Serum pro-inflammatory cytokines IL-1β, IL-6, and TNF-α were sharply increased after DSS modeling. BLE moderately inhibited inflammatory factor secretion, while BLE exerted stronger anti-inflammatory activity in a dose-dependent fashion (Figure 1G). Splenic flow cytometry was performed to assess Treg and Th17 cell subsets separately. The DSS challenge markedly reduced the proportion of CD4^+^Foxp3^+^Tregs and elevated the percentage of CD4^+^IL-17A^+^Th17 cells in spleen. Treatment with triple BLE only slightly reversed these abnormal changes, while both low- and high-dose BLEB significantly upregulated Treg frequency, suppressed Th17 accumulation, and restored the Treg/Th17 ratio, showing much stronger regulatory effects on individual T-cell populations (Figure 1H). All macroscopic, histological, and immune phenotypic data collectively proved that the quadruple BLEB formula containing *B. cereus* possessed superior protective capacity compared with triple BLE.

### 3.2. BLEB Dose-Dependently Repairs Damaged Intestinal Epithelial Barrier

qRT-PCR results showed that DSS intervention significantly downregulated the mRNA levels of tight junction and mucin genes including E-cadherin, Occludin, MUC-2, and Claudin-1. Both BLEB-L and BLEB-H notably upregulated the transcription of these barrier-related genes, with the high-dose group displaying the strongest regulatory effect (Figure 2A). Western blot analysis of corresponding protein expression yielded consistent trends. DSS obviously suppressed the protein abundance of E-cadherin, Occludin, MUC-2, and Claudin-1, while BLEB dose-dependently restored these key barrier proteins to maintain mucosal integrity (Figure 2B).

### 3.3. Dynamic Reshaping of Gut Microbial Composition by BLEB at Day 4 and Day 8

Dynamic 16S rRNA sequencing was performed on days 4 and 8 to characterize BLEB mediated gut microbiota remodeling. DSS treatment significantly reduced the Chao1 α-diversity index at these two time points, manifested as a significant loss of microbial richness. BLEB intervention partially recovered microbial richness at both time points, without obvious time-dependent improvement from day 4 to day 8 (Figure 3A,E). PCA revealed that the separation between the DSS group and BLEB intervention groups was more pronounced at day 8 than day 4, indicating that continuous BLEB treatment induced more substantial reshaping of the overall gut microbial community over time (Figure 3B,F). At genus and species levels, DSS reduced the relative abundance of beneficial *Lactobacillus* while enriching conditional pathogens. BLEB dose-dependently elevated the proportion of protective *Lactobacillus* at both time points, and this enrichment effect was enhanced on day 8 (Figure 3C,D,G,H). These results suggested that BLEB induced continuous, favorable reconstruction of dysbiotic gut microbiota in colitis mice.

### 3.4. Untargeted Metabolomics Identifies Spermidine as the Core Protective Metabolite Regulated by BLEB

Untargeted LC-MS metabolomics of intestinal contents was used to screen differential metabolites responding to BLEB intervention. PCA of intestinal metabolic profiles displayed clear segregation between the Con and DSS groups, while BLEB-L and BLEB-H samples distributed between the two clusters, demonstrating that BLEB partially normalized DSS-disturbed metabolic phenotypes (Figure 4A). Volcano plots illustrated massive metabolite shifts triggered by colitis: 376 metabolites were upregulated and 279 were downregulated in DSS relative to Con; compared with the DSS group, 300 metabolites rose and 227 declined after low-dose BLEB treatment, while high-dose BLEB induced 330 upregulated and 188 downregulated differential metabolites (Figure 4B). KEGG bubble enrichment revealed prominent disturbances in nitrogen metabolism, pentose phosphate pathway, glutathione metabolism, and multiple amino acid and lipid metabolic pathways upon DSS exposure. After BLEB intervention, carbohydrate metabolism, amino acid metabolism, and antioxidant-related pathways were largely recovered (Figure 4C). Heatmap visualization of characteristic differential metabolites showed obvious depletion of polyamine substances including spermidine, spermidine-C4:0, and cadaverine in the DSS group, alongside disordered levels of L-phenylalanine, *N*-α-acetyl-L-citrulline and other amino acid derivatives; both doses of BLEB effectively restored the abundance of these polyamine metabolites (Figure 4D). Spearman correlation analysis further uncovered tight correlations between core polyamines and gut microbial taxa. Key protective metabolites such as spermidine and cadaverine were significantly positively correlated with beneficial genera within the family *Lactobacillaceae*, including *Lactobacillus*, *Limosilactobacillus*, and *Ligilactobacillus*, while negatively correlated with opportunistic genera including *Helicobacter* and *Lachnoclostridium* (Figure 4E). Collectively, these metabolomic data confirmed that BLEB reshapes overall intestinal metabolic homeostasis, and elevates endogenous spermidine and related polyamines by enriching beneficial lactobacilli, forming a microbiota–polyamine regulatory axis to alleviate colonic injury.

### 3.5. Transcriptomic Profiling of DSS-Injured Caco-2 Epithelial Cells Reveals Gene-Regulatory Responses upon Spermidine Treatment

An in vitro DSS-induced Caco-2 epithelial injury model combined with RNA-seq transcriptomic profiling was constructed to explore potential molecular responses of intestinal epithelial cells to spermidine (SPD). PCA results exhibited clear transcriptional segregation across the control, DSS injury, and DSS + SPD co-treatment groups, manifesting profound gene expression remodeling triggered by DSS and spermidine respectively (Figure 5A). Differential gene analysis showed 2015 upregulated and 2116 downregulated genes in DSS-treated cells relative to normal controls; comparison between the SPD intervention group and DSS group identified 135 significantly upregulated and 179 downregulated transcripts, suggesting spermidine could partially reverse DSS-provoked transcriptional perturbations (Figure 5B). KEGG enrichment analysis indicated that genes perturbed by DSS were mainly enriched in carbon metabolism, glutathione metabolism, oxidative phosphorylation, and amino metabolic pathways. After spermidine supplementation, enriched pathways centered on steroid hormone biosynthesis, PPAR signaling, glutathione metabolism, oxidative phosphorylation, and cytochrome P450 xenobiotic metabolism (Figure 5C). Hierarchical-clustering heatmaps further confirmed that spermidine partially restored the abnormal expression of core genes disturbed by DSS exposure (Figure 5D). Taken together, cellular transcriptomic data reveal that spermidine elicits broad gene-expression changes involving oxidative stress, energy-metabolism and lipid-metabolism-related signaling cascades in DSS-challenged Caco-2 cells.

## 4. Discussions

In this study, we systematically compared the anti-colitis efficacy of triple-oxygen-sensitive-probiotic mixture (BLE) and four-strain formulation BLEB supplemented with facultative aerobic *B. cereus* in an acute DSS-induced colitis mouse model, and uncovered a potential microbiota–spermidine-related metabolic axis that may partially underpin the superior therapeutic effect of BLEB. A previous study has confirmed that quadruple probiotics containing aerobic strains relieve chronic colitis [20]. Our work extended this protective phenotype to acute intestinal inflammatory injury and supplemented comprehensive dose-dependent phenotypic data. Most critically, we excavated a deeper downstream metabolic regulatory pathway entirely unexplored in the earlier study. While the prior research only conducted simple phenotypic and single-timepoint microbial analysis, we integrated dynamic dual-timepoint microbiota sequencing, untargeted metabolomics, and epithelial cell transcriptomics to identify spermidine as the core functional metabolite connecting reshaped gut microbiota and mucosal repair, which provides an unprecedented metabolic mechanism to interpret the synergistic advantage conferred by the addition of *B. cereus*.

Macroscopic observation, body weight dynamics, DAI scoring, colon length measurement, and H&E histopathological staining collectively validated that triple BLE only yielded mild relief against DSS-triggered colonic lesions, whereas low- and high-dose BLEB exerted robust anti-colitis effects in a clear dose-dependent manner (Figure 1). The poor performance of BLE stems from the high oxygen sensitivity of its three constituent strains: *B. infantis*, *L. acidophilus*, and *E. faecali* suffer substantial viability *loss* when passing through the oxygen-rich stomach and small intestine, greatly reducing their gut colonization ability [26,27,28]. The gastrointestinal tract presents a gradient of oxygen tension that progressively declines from the proximal gut to the colon, creating an aerobic barrier that impairs the survival of strictly anaerobic probiotic strains before they reach their target colonic niche [29]. By contrast, facultative aerobic *B. cereus* in BLEB scavenges intestinal free oxygen and creates an anaerobic niche to protect oxygen-sensitive probiotic strains during gastrointestinal transit [19]. This unique ecological synergy improves the survival and colonization of bifidobacteria and lactobacilli in vivo, which is supported by our dynamic 16S rRNA sequencing results showing that sustained BLEB treatment dose-dependently increased the relative abundance of beneficial *Lactobacillus*, accompanied by progressive remodeling of overall gut microbial community structure over time (Figure 3). Consistent with this microbial remodeling, BLEB intervention reduced serum pro-inflammatory IL-1β, IL-6, and TNF-α, partially restored splenic Treg/Th17 cell balance, and rescued the expression of intestinal tight-junction-related proteins. It should be noted that our in-vivo data were obtained from an acute chemically triggered DSS colitis mouse model. Human UC represents a heterogeneous chronic inflammatory disorder shaped by complex genetic and environmental factors. Differences in gut microbiota composition, metabolic signatures and inflammatory markers exist between acute murine DSS injury and clinical UC, and direct translational extrapolation from mouse findings to human patients should be exercised with caution. Further validation using chronic colitis animal models and human clinical cohorts will be required in future work.

Building on the observed microbial reshaping driven by BLEB, untargeted metabolomics enabled us to probe downstream functional metabolites that mediate mucosal protection. Polyamines, represented by spermidine, are key microbiota-derived signaling molecules that control intestinal epithelial renewal, tight junction maintenance, and local immune homeostasis [21,30]. The colonic polyamine pool largely depends on the biosynthetic ability of symbiotic bacteria, rather than dietary sources, as most dietary polyamines are absorbed by the small intestine [31]. An increasing amount of evidence suggests that ecological dysbiosis during colitis often reduces the production of polyamines by commensal bacteria, leading to depletion of luminal spermidine and the formation of a self-reinforcing pathological cycle characterized by metabolic product deficiency, barrier damage, and exacerbation of inflammation [25]. In our study, Spearman correlation analyses supported a potential mechanistic link between enriched members of the *Lactobacillaceae* family (including *Lactobacillus* and *Ligilactobacillus*) and elevated polyamine abundance, consistent with prior reports identifying lactobacilli as major intestinal spermidine producers in the gut ecosystem [32]. Notably, most previous probiotic–colitis studies mainly focused on short-chain fatty acids as effector metabolites, whereas the microbiota–polyamine axis remains comparatively under-explored for compound probiotic formulations containing *B. cereus*. Our findings highlight that the beneficial outcome of BLEB is not merely attributed to direct anti-inflammatory effects of probiotic strains themselves; instead, remodeling of microbial community shifts metabolic output toward spermidine-dominated polyamine production, which may act as a key intermediate mediator linking probiotic intervention to mucosal repair. In the present multi-omics profiling, we only analyzed samples from Con, DSS, BLEB-L and BLEB-H groups. Multi-omics datasets for the BLE group and an independent single-strain *B. cereus* intervention group were not included, which prevents us from fully disentangling the individual contribution of *B. cereus* from the composite protective effects of the four-strain BLEB formulation.

Given the complex in vivo microenvironment, it remains difficult to dissect whether elevated spermidine can directly exert protective effects on intestinal epithelial cells in mice. To explore cell-autonomous molecular responses to spermidine, we employed DSS-injured Caco-2 epithelial cells combined with RNA-seq transcriptomic profiling. Our cellular transcriptomic data demonstrated that spermidine intervention could reverse large-scale DSS-provoked transcriptional perturbations in intestinal epithelial cells. KEGG enrichment analysis revealed multiple altered functional modules upon spermidine treatment, including glutathione-dependent antioxidant defense, PPAR signaling, and cytochrome P450-mediated xenobiotic metabolism. Among these enriched pathways, we focused on glutathione-dependent antioxidant defense because corresponding core genes were displayed in our heatmap. Within the glutathione-metabolism pathway, DSS exposure triggered transcriptional activation of multiple glutathione-synthesis and glutathione-utilizing genes including GCLM, GSTM family members, and GPX2, representing an intrinsic cellular defensive response triggered by DSS-induced oxidative stress [33,34]. DSS-provoked oxidative stress evokes endogenous antioxidant transcriptional programs; spermidine further amplifies the transcription of these glutathione-associated genes, which may contribute to enhanced cellular antioxidant-related responses against DSS-induced epithelial injury [35]. Although our transcriptome did not show prominent transcriptional changes in SLC7A11, this augmented glutathione-related transcriptional program still has the potential to lower cellular susceptibility to oxidative and lipid-peroxide-driven cell death, consistent with published observations that spermidine reinforces glutathione-centered antioxidant defense under epithelial injury [23]. Collectively, transcriptional modulation of glutathione-metabolism gene programs raises the possibility that spermidine may counteract DSS-triggered epithelial injury partially by reprogramming oxidative-stress-related transcriptional signatures.

Several additional limitations of the current study should be acknowledged. First, the microbiota–spermidine regulatory axis is inferred based on 16S rRNA sequencing, untargeted metabolomics, and Spearman correlation analysis in vivo. Correlative multi-omics data cannot fully establish strict causal relationships. Further intervention-based studies, such as germ-free or antibiotic-depleted mouse models, would help to definitively dissect the causal interplay between BLEB-reshaped gut microbiota and elevated luminal spermidine. Second, our in vitro spermidine protection results are obtained from the Caco-2 cell line; organoid or ex vivo colon tissue culture systems together with biochemical oxidative-stress or ferroptosis markers would offer a more physiologically relevant platform to validate spermidine-mediated epithelial protective function. Moreover, spermidine levels are semi-quantitative relative intensities from untargeted metabolomics, which awaits future targeted-metabolomics absolute-quantification validation.

Collectively, our study demonstrates that supplementation of facultative aerobic *B. cereus* markedly enhances the anti-colitis capacity of the triple-anaerobic-probiotic mixture BLE. The quadruple-probiotic BLEB exerts multi-layered intestinal protection, including alleviation of systemic inflammation, restoration of Treg/Th17 immune balance, and preservation of intestinal epithelial barrier integrity. The superior activity of BLEB is partially attributed to the oxygen-scavenging ecological advantage of *B. cereus*, which facilitates the survival and colonization of oxygen-sensitive anaerobic probiotic strains, drives favorable gut microbiota remodeling, and consequently elevates intestinal endogenous spermidine abundance (Figure 6). This work provides dose-dependent in vivo evidence and a novel microbiota–spermidine metabolic mechanism for compound probiotic formulations containing aerobic strains, and offers practical references for rational optimization of probiotic combinations for IBD intervention.

## 5. Conclusions

Taken together, supplementation of facultative-aerobic *B. cereus* substantially improves the anti-colitis efficacy of the triple-probiotic preparation BLE in DSS-challenged mice. Correlative multi-omics data indicate that the enhanced protective effect of quadruple-probiotic BLEB may be partially associated with gut microbiota remodeling and elevated luminal spermidine abundance. Our transcriptomic findings indicate that spermidine may counteract epithelial injury partly by potentiating glutathione-centered antioxidant transcriptional responses. This study highlights the ecological synergy between facultative-aerobic and oxygen-sensitive probiotic strains and identifies a correlative microbiota–spermidine axis potentially contributing to mucosal protection, providing new mechanistic insights and pre-clinical evidence for developing optimized probiotic therapies for ulcerative colitis.

## Figures and Tables

**Figure 1:**
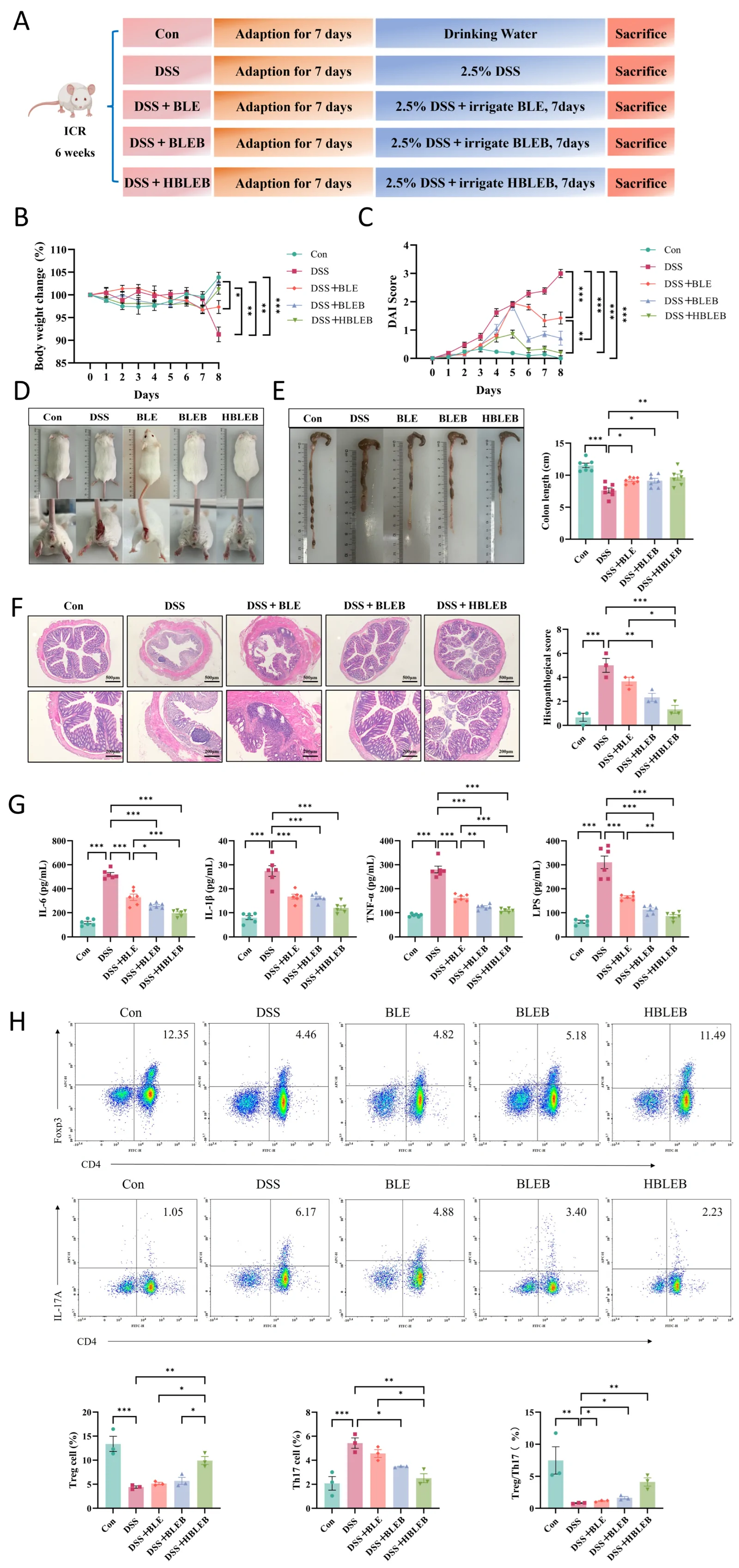
BLEB alleviated DSS-induced acute colitis in ICR mice in a dose-dependent manner. (**A**) Schematic diagram of the animal experimental design. Six-week-old male ICR mice were acclimatized for 7 days, followed by 2.5% DSS drinking water administration combined with intragastric gavage of BLE, low-dose BLEB (BLEB-L), or high-dose BLEB (BLEB-H) for 7 consecutive days. Mice in the Con group received normal drinking water and vehicle gavage. All animals were sacrificed at the experimental endpoint. (**B**) Dynamic changes in body weight among different groups (*n* = 6 per group). (**C**) Disease activity index (DAI) scores recorded throughout DSS-colitis progression (*n* = 6 per group). (**D**) Representative macroscopic appearances of mice and colonic gross morphology. (**E**) Representative colon photographs and quantitative measurement of colon length (*n* = 7 per group). (**F**) Representative H&E-stained colonic tissue sections (scale bar: 500 μm and 200 μm) and corresponding histopathological injury scores (*n* = 3 per group). (**G**) Serum concentrations of pro-inflammatory cytokines IL-6, IL-1β, TNF-α, and LPS detected by ELISA (*n* = 6 per group). (**H**) Representative flow cytometry dot plots and statistical analysis of splenic CD4^+^Foxp3^+^ Treg cells, CD4^+^IL-17A^+^ Th17 cells, and the Treg/Th17 ratio (*n* = 3 per group). Data are presented as mean ± SEM. * *p* < 0.05, ** *p* < 0.01, *** *p* < 0.001. Con, control group; DSS, dextran sulfate sodium-treated group; BLE, triple-strain anaerobic probiotic mixture; BLEB-L, low-dose BLEB; BLEB-H, high-dose BLEB.

**Figure 2:**
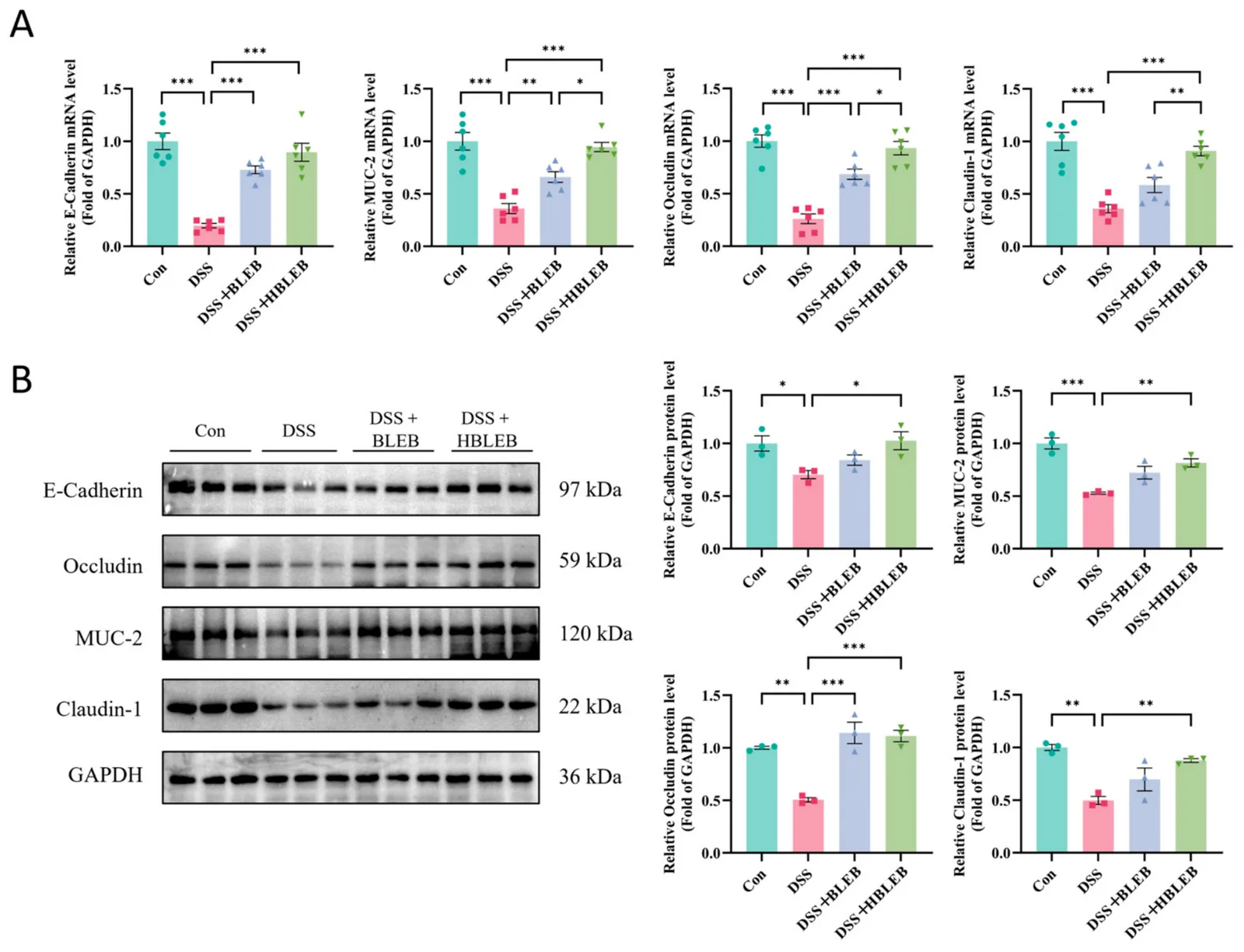
BLEB restored the expression of intestinal mucosal barrier-associated genes and proteins in DSS-colitis mice. (**A**) qRT-PCR analysis of mRNA expression levels of epithelial barrier-related genes, including E-Cadherin, MUC-2, Occludin, and Claudin-1. GAPDH was used as the internal reference gene (*n* = 6 per group). (**B**) Representative Western blot bands and quantitative analysis for E-Cadherin, MUC-2, Occludin, and Claudin-1 protein levels. GAPDH served as the loading control (*n* = 3 per group). Data are presented as mean ± SEM. * *p* < 0.05, ** *p* < 0.01, *** *p* < 0.001. Con, control group; DSS, dextran sulfate sodium-treated group; BLEB-L, low-dose BLEB; BLEB-H, high-dose BLEB.

**Figure 3:**
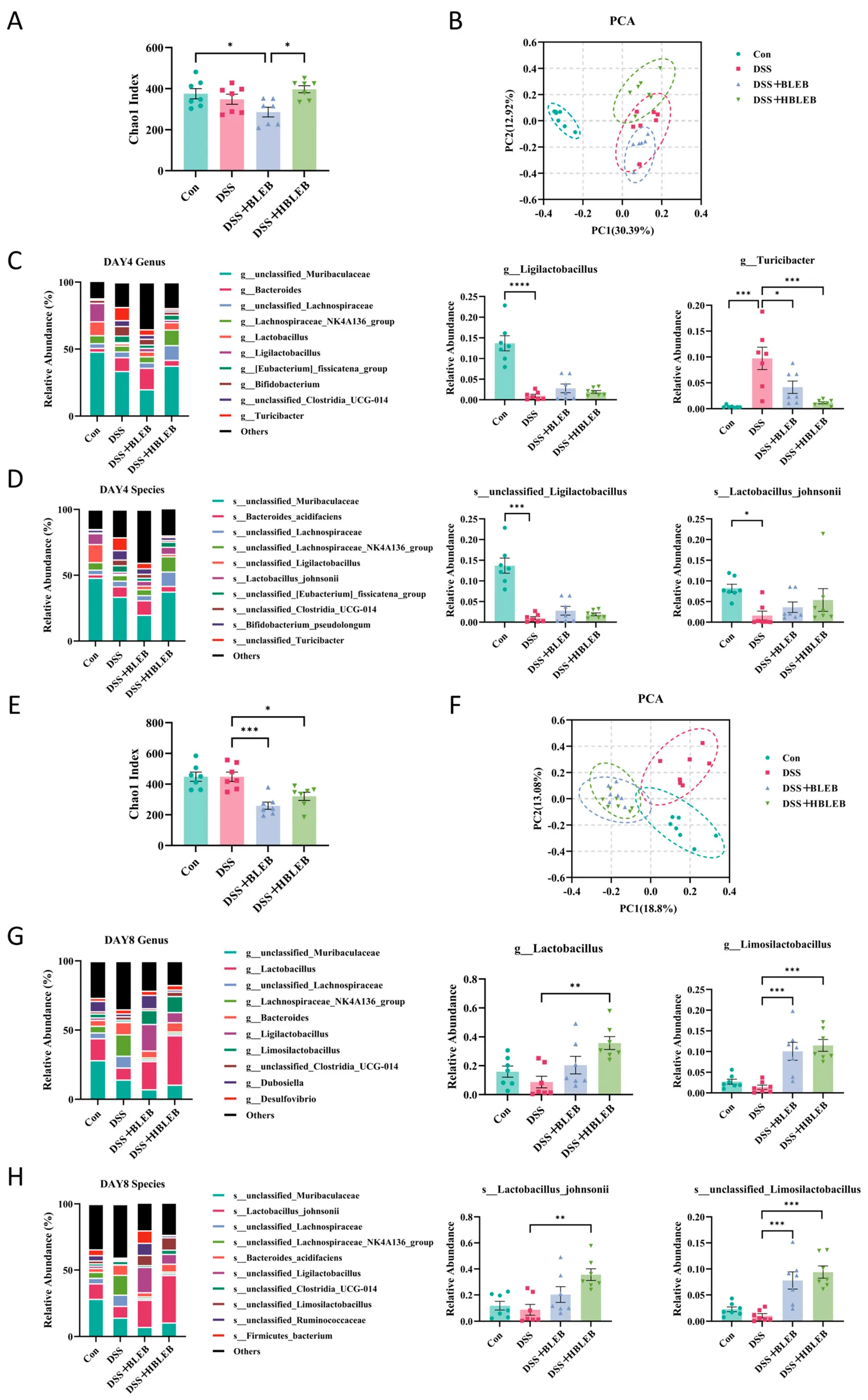
BLEB reshaped gut microbiota profiles in DSS-induced colitis mice at day 4 and day 8. (**A**,**E**) Alpha diversity (Chao1 index) of gut microbiota at day 4 (**A**) and day 8 (**E**). (**B**,**F**) Principal component analysis (PCA) based on 16S rRNA sequencing data at day 4 (**B**) and day 8 (**F**). (**C**,**G**) Relative-abundance bar charts at the genus level and statistical quantification of representative bacterial genera at day 4 (**C**) and day 8 (**G**). (**D**,**H**) Species-level relative-abundance bar charts and statistical quantification of representative bacterial species at day 4 (**D**) and day 8 (**H**). Data are presented as mean ± SEM (*n* = 7 per group). * *p* < 0.05, ** *p* < 0.01, *** *p* < 0.001, **** *p* < 0.0001. Con, control group; DSS, dextran sulfate sodium-treated group; BLEB-L, low-dose BLEB; BLEB-H, high-dose BLEB.

**Figure 4:**
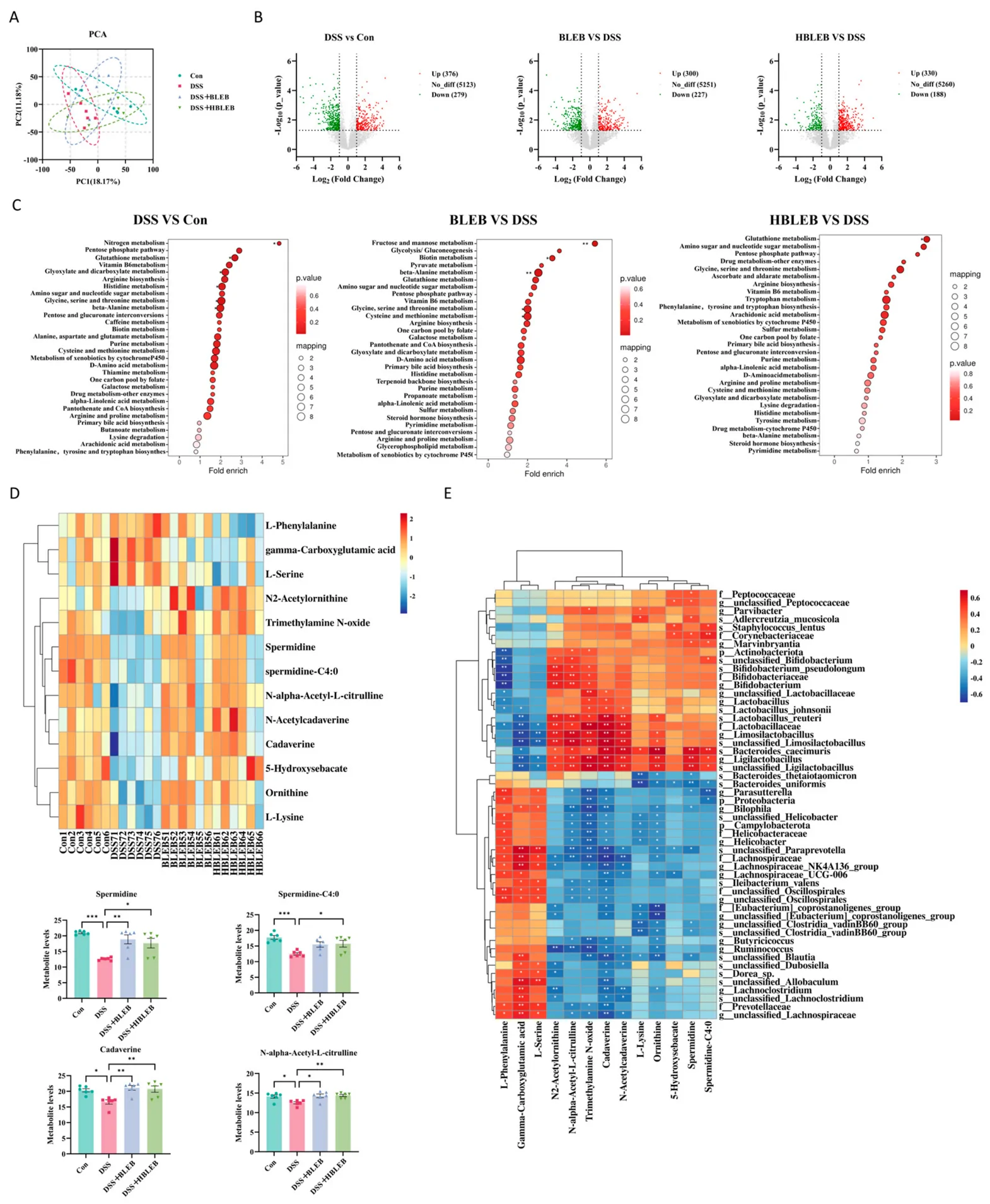
BLEB remodels intestinal metabolite profiles and restores polyamine-related metabolites in DSS-colitis mice. (**A**) PCA score plot of untargeted LC-MS metabolomics derived from intestinal contents across all experimental groups. (**B**) Volcano plots displaying differential metabolites for DSS vs. Con, BLEB-L vs. DSS, and BLEB-H vs. DSS comparisons. (**C**) KEGG bubble enrichment analysis of differential metabolites from each comparison group. (**D**) Heatmap of core differential metabolites and quantitative analysis of representative polyamine metabolites including spermidine, spermidine-C4:0, cadaverine, and *N*-alpha-Acetyl-L-citrulline. (**E**) Hierarchical-clustering heatmap showing the overall alteration of differential intestinal metabolites among groups. Data are presented as mean ± SEM (*n* = 6 per group). * *p* < 0.05, ** *p* < 0.01, *** *p* < 0.001. Con, control group; DSS, dextran sulfate sodium-treated group; BLEB-L, low-dose BLEB; BLEB-H, high-dose BLEB.

**Figure 5:**
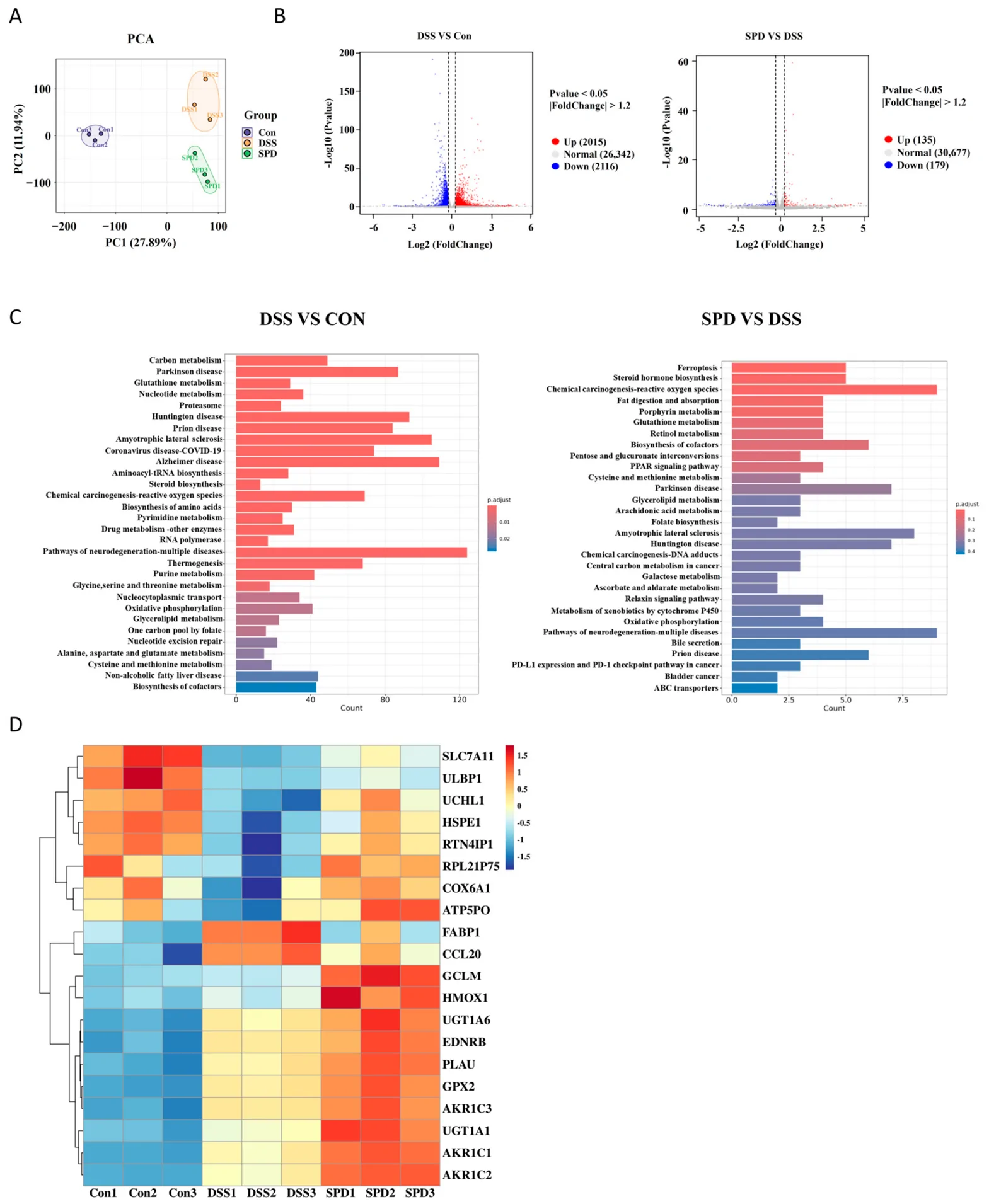
Spermidine reprograms transcriptomic profiles in DSS-injured Caco-2 epithelial cells. (**A**) PCA score plot based on RNA-seq transcriptome data of Caco-2 cells among Con, DSS, and SPD groups (*n* = 3 per group). (**B**) Volcano plots illustrating differentially expressed genes (DEGs) for DSS vs. Con and SPD vs. DSS comparisons. Red dots represent significantly upregulated genes, blue dots represent significantly downregulated genes, and gray dots denote genes with no significant difference. (**C**) KEGG enrichment analysis of differentially expressed genes in DSS versus Con and SPD versus DSS groups. (**D**) Hierarchical-clustering heatmap displaying the expression patterns of core differentially expressed genes across all samples. Con, control group; DSS, DSS-treated Caco-2 cells; SPD, spermidine intervention group.

**Figure 6:**
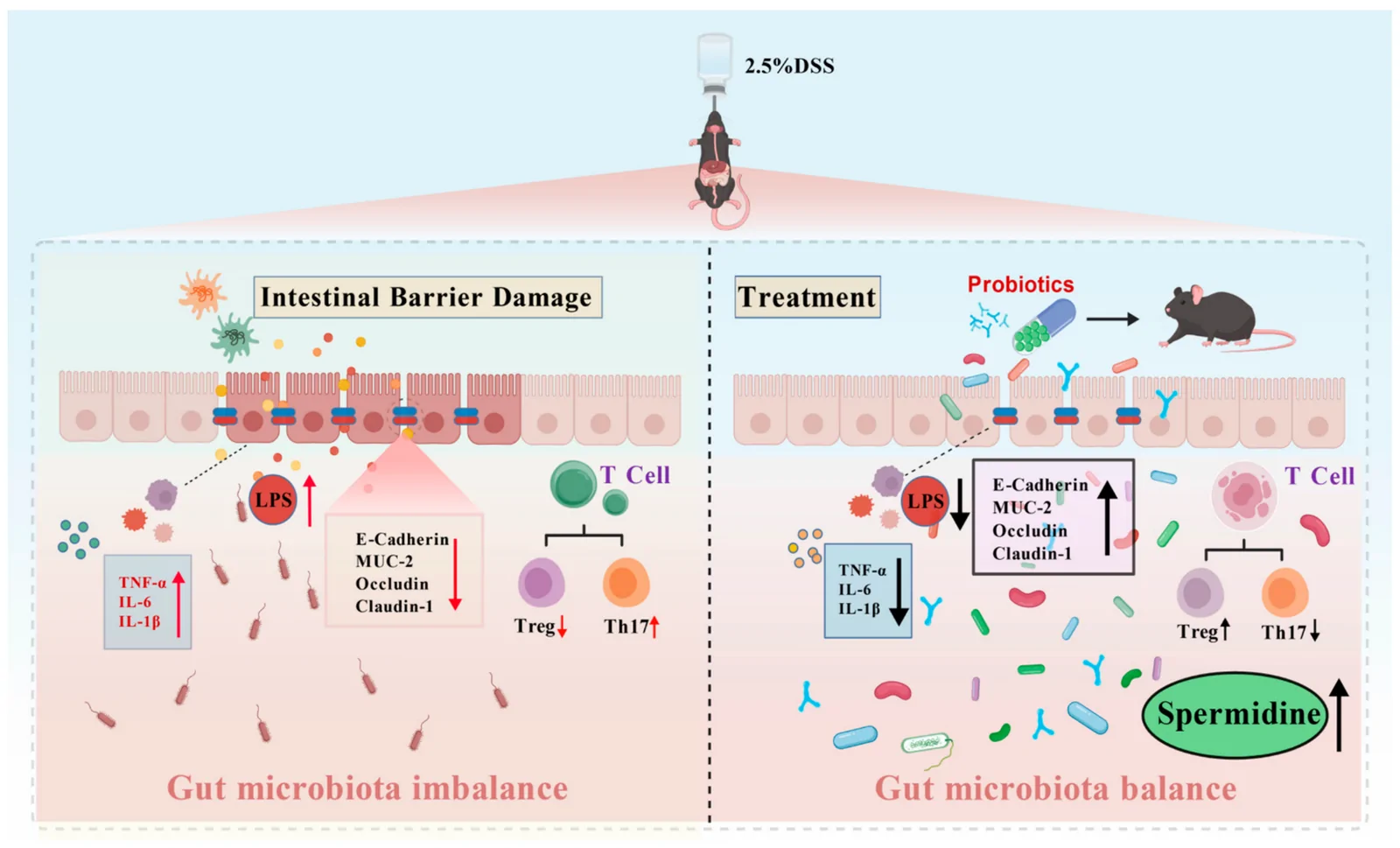
Schematic illustration summarizing potential protective effects of BLEB against DSS-induced ulcerative colitis. DSS exposure triggers intestinal barrier injury, gut microbiota dysbiosis, excessive release of pro-inflammatory cytokines (TNF-α, IL-6, IL-1β), and Treg/Th17 immune imbalance in mice. As a four-strain probiotic formulation, BLEB may remodel gut microbiota and increase luminal spermidine levels (supported by correlative multi-omics data). BLEB restores intestinal tight-junction-related protein expression, suppresses pro-inflammatory cytokine release and rescues Treg/Th17 homeostasis, which collectively could alleviate colonic inflammatory injury (Created with BioGDP.com [36]).

## Data Availability

The datasets generated during and/or analyzed during the current study are deposited in the GenBank Sequence Read Archive with the accession number PRJNA1515471.

## Abbreviations

ANOVA	Analysis of variance
BLE	*Bifidobacterium infantis*, *Lactobacillus acidophilus*, *Enterococcus faecalis* probiotic mixture
BLEB	BLE supplemented with facultative-aerobic *Bacillus cereus* quadruple probiotic formulation
BLEB-L	Low-dose BLEB
BLEB-H	High-dose BLEB
cDNA	Complementary deoxyribonucleic acid
Con	Control group
DAI	Disease activity index
DEGs	Differentially expressed genes
DSS	Dextran sulfate sodium
ELISA	Enzyme-linked immunosorbent assay
GAPDH	Glyceraldehyde-3-phosphate dehydrogenase
GPX2	Glutathione peroxidase 2
H&E	Hematoxylin–eosin
IBD	Inflammatory bowel disorder
KEGG	Kyoto Encyclopedia of Genes and Genomes
LPS	Lipopolysaccharide
PCA	Principal component analysis
PVDF	Polyvinylidene difluoride
qRT-PCR	Quantitative real-time polymerase chain reaction
SEM	Standard error of the mean
SLC7A11	Solute carrier family 7 member 11
SPD	Spermidine
Treg	Regulatory T cell
Th17	T helper 17 cell
UC	Ulcerative colitis

## References

[1] Zhao M., Zhou H., Jiang R., Gao Y., Zhang P., Wang F. (2026). Colon-targeted alginate/chitosan microspheres for co-delivery of synbiotics and nanozymes ameliorate inflammatory bowel disease. Food Res. Int..

[2] Allwang M., Wipplinger M., Akbarimoghaddam P., Alonso-Roman R., Cseresnyes Z., Dietschmann A., Wegner V., Feile A., Bachelot Y., Warschinke M. (2026). Human Colitis-on-Chip Model Reveals Dual Roles of Butyrate in Epithelial and Macrophage Defense Against Candida albicans Tissue Invasion. Small.

[3] Rubin D.T., Ananthakrishnan A.N., Siegel C.A., Barnes E.L., Long M.D. (2025). ACG Clinical Guideline Update: Ulcerative Colitis in Adults. Am. J. Gastroenterol..

[4] Xue J.C., Hou X.T., Zhao Y.W., Yuan S. (2025). Biological agents as attractive targets for inflammatory bowel disease therapeutics. Biochim. Biophys. Acta Mol. Basis Dis..

[5] Oliveira J.T., Pieniz S. (2026). Role of creatine supplementation in intestinal health: A narrative review of its antioxidant and anti-inflammatory potential. Nutrition.

[6] Kayal M., Shah S. (2019). Ulcerative Colitis: Current and Emerging Treatment Strategies. J. Clin. Med..

[7] Awoniyi M., El Hag M., Hernandez J., Yang Q., Evans N., Nemet I., Ngo B., Coskuner D., Zhou J., Farmer M. (2026). Dysbiotic microbiota trigger colitis-associated colorectal cancer and imprint a distinctive bile acid profile in a PSC-IBD model. Gut.

[8] Rosali M.I., V. Thanga Velu D.P., Mokhtar M.H., Raja Ali R.A., Mohd Mokhtar N., A. Hamid A. (2026). Specificity vs. Synergy Between Single-Strain and Multi-Strain Probiotics for Ulcerative Colitis Treatment: A Review of the Literature. Biomedicines.

[9] Luo W., Zhuang Y., Shen S., Wang Y., Zhu Y., Liu Y., Zhang S., Zhang J., Li S., Zhong L. (2026). Eupalinolide B alleviates ulcerative colitis by targeting HMGB1 through modulation of the HMGB1/TLR4/MyD88/NF-κB signaling pathway. Phytomedicine.

[10] Miao R., Wang S., Yin H., Zhu R., Yin Y., Liao W., Wang S., Zhang J., Li R., Xu J. (2026). Scutellaria baicalensis-Derived Extracellular Vesicles Alleviate Inflammatory Bowel Disease by Inhibiting the NF-κB/NLRP3 Pathway. Int. J. Nanomed..

[11] Li M., Chen D., Shao Y., Wang Z., Peng Y., Zhao Q., Liu L. (2026). Sinomenine alleviates ulcerative colitis by targeting FXR to regulate arachidonic acid metabolism and Th17/Treg homeostasis. Eur. J. Pharmacol..

[12] Giudice A., Brescia C., Morano D., Viglietto G., Luzza F., Amato R., Spagnuolo R. (2026). Th17/treg balance in Inflammatory Bowel Disease: The role of microbial, and genetic regulators in disease modulation. Front. Cell Dev. Biol..

[13] Park N., Lee B., Jeon H.J., Yeon Kim J., Park S., Kim S.E., Lee D.K., Lee Y. (2026). Inulin-Butyrate Nanogel for Modulation of Gut Microbiome, Intestinal Barrier, and Regulatory T-Cells in Colitis. Small.

[14] Liu A., Zhu X.J., Sun W.D., Bi S.Z., Zhang C.Y., Li J.H. (2026). Nicotinamide N-methyltransferase in inflammatory bowel disease: Multidimensional regulation, mechanistic insights, and therapeutic potential. Exp. Cell Res..

[15] Rodríguez E., Peirotén Á., Landete J.M., Medina M., Arqués J.L. (2015). Gut Catalase-Positive Bacteria Cross-Protect Adjacent Bifidobacteria from Oxidative Stress. Microbes Env..

[16] Portela C.A., Smart K.F., Tumanov S., Cook G.M., Villas-Bôas S.G. (2014). Global metabolic response of Enterococcus faecalis to oxygen. J. Bacteriol..

[17] Chen Y., Wang S., Chen A., Lin Z., Wang H., Li W., Liu J., Yao J., Tian D., Lei Y. (2026). Multi-omics Analysis Reveals the Protection of a Quadruple Probiotic Mixture in Experimental Autoimmune Hepatitis. Probiotics Antimicrob. Proteins.

[18] Jiang C., Shi J., Zhuang X., Zhou M., Zhong H. (2025). Effects of a combination of bifidobacteria quadruple viable bacteria tablets and quadruple therapy on inflammatory response and Helicobacter pylori eradication rate in patients with Helicobacter pylori positive gastric ulcers. Front. Med..

[19] Sheng K., Xu Y., Kong X., Wang J., Zha X., Wang Y. (2021). Probiotic Bacillus cereus Alleviates Dextran Sulfate Sodium-Induced Colitis in Mice through Improvement of the Intestinal Barrier Function, Anti-Inflammation, and Gut Microbiota Modulation. J. Agric. Food Chem..

[20] Chen Y., Zhang L., Hong G., Huang C., Qian W., Bai T., Song J., Song Y., Hou X. (2020). Probiotic mixtures with aerobic constituent promoted the recovery of multi-barriers in DSS-induced chronic colitis. Life Sci..

[21] Fang X., Guo Y., Huang J., Zhang M. (2026). Polyamines as a Universal Language of Host-Microbiota Symbiosis. Research.

[22] Zhou X., Xu Q., Zhang X., Wang H., Bai Y., Wu Y., Liu X., Wang Z., Hu J., Huang M. (2024). Mucin alleviates colonic barrier dysfunction by promoting spermine accumulation through enhanced arginine metabolism in *Limosilactobacillus mucosae*. mSystems.

[23] Zhang M., Dou Z., Yang L., Wang H., Tian B., Hong S., Yang Q., Lu J., Chen X. (2026). Ferroptosis inhibition by spermidine via the NCOA4/Ferritin axis and polyamine-microbiota dysbiosis in ulcerative colitis. J. Nutr. Biochem..

[24] Carriche G.M., Almeida L., Stüve P., Velasquez L., Dhillon-LaBrooy A., Roy U., Lindenberg M., Strowig T., Plaza-Sirvent C., Schmitz I. (2021). Regulating T-cell differentiation through the polyamine spermidine. J. Allergy Clin. Immunol..

[25] Niechcial A., Schwarzfischer M., Wawrzyniak M., Atrott K., Laimbacher A., Morsy Y., Katkeviciute E., Häfliger J., Westermann P., Akdis C.A. (2023). Spermidine Ameliorates Colitis via Induction of Anti-Inflammatory Macrophages and Prevention of Intestinal Dysbiosis. J. Crohns Colitis.

[26] Tsuji H., Oozeer R., Matsuda K., Matsuki T., Ohta T., Nomoto K., Tanaka R., Kawashima M., Kawashima K., Nagata S. (2012). Molecular monitoring of the development of intestinal microbiota in Japanese infants. Benef. Microbes.

[27] Olsson L.M., Boulund F., Nilsson S., Khan M.T., Gummesson A., Fagerberg L., Engstrand L., Perkins R., Uhlén M., Bergström G. (2022). Dynamics of the normal gut microbiota: A longitudinal one-year population study in Sweden. Cell Host Microbe.

[28] Talwalkar A., Kailasapathy K. (2004). The role of oxygen in the viability of probiotic bacteria with reference to *L. acidophilus* and *Bifidobacterium* spp. Curr. Issues Intest. Microbiol..

[29] Kint N., Alves Feliciano C., Martins M.C., Morvan C., Fernandes S.F., Folgosa F., Dupuy B., Texeira M., Martin-Verstraete I. (2020). How the Anaerobic Enteropathogen Clostridioides difficile Tolerates Low O_2_ Tensions. mBio.

[30] Nakamura A., Matsumoto M. (2025). Role of polyamines in intestinal mucosal barrier function. Semin. Immunopathol..

[31] Kitada Y., Muramatsu K., Toju H., Kibe R., Benno Y., Kurihara S., Matsumoto M. (2018). Bioactive polyamine production by a novel hybrid system comprising multiple indigenous gut bacterial strategies. Sci. Adv..

[32] Chen Y., Fang J.Y. (2025). The role of colonic microbiota amino acid metabolism in gut health regulation. Cell Insight.

[33] Lin Y., Zheng X., Chen J., Luo D., Xie J., Su Z., Huang X., Yi X., Wei L., Cai J. (2019). Protective Effect of *Bruguiera gymnorrhiza* (L.) Lam. Fruit on Dextran Sulfate Sodium-Induced Ulcerative Colitis in Mice: Role of Keap1/Nrf2 Pathway and Gut Microbiota. Front. Pharmacol..

[34] Din A.U., Hassan A., Zhu Y., Zhang K., Wang Y., Li T., Wang Y., Wang G. (2020). Inhibitory effect of Bifidobacterium bifidum ATCC 29521 on colitis and its mechanism. J. Nutr. Biochem..

[35] Xi X., Li J., Wang Y., Ni Y., Zhou J., Cao L., Wei W., Gao J. (2026). Codonopsis pilosula as the sovereign herb in Shenling Baizhu formula ameliorates DSS-induced ulcerative colitis via spermidine-AMPK-mediated mitophagy to inhibit the cGAS-STING inflammatory axis. Int. Immunopharmacol..

[36] Jiang S., Li H., Zhang L., Mu W., Zhang Y., Chen T., Wu J., Tang H., Zheng S., Liu Y. (2025). Generic Diagramming Platform (GDP): A comprehensive database of high-quality biomedical graphics. Nucleic Acids Res..

